# Safety and efficacy of noninvasive ventilation in patients with blunt chest trauma: a systematic review

**DOI:** 10.1186/cc12821

**Published:** 2013-07-22

**Authors:** Abhijit Duggal, Pablo Perez, Eyal Golan, Lorraine Tremblay, Tasnim Sinuff

**Affiliations:** 1 Medical Intensive Care Unit, Respiratory Institute, Cleveland Clinic Foundation, Euclid Avenue, Cleveland, OH 44195, USA; 2 Department of Critical Care Medicine, Sunnybrook Health Sciences Centre and University of Toronto, Bayview Avenue, Toronto, ON, M4N 3M5, Canada; 3 Interdepartmental Division of Critical Care, University of Toronto and Sunnybrook Research Institute, Sunnybrook Health Sciences Centre, Bayview Avenue, Toronto, ON, M4N 3M5, Canada; 4 Department of Medicine, University Health Network, Bathhurst Street, Toronto, ON, M5T 2S8, Canada; 5 Department of Surgery, Sunnybrook Health Sciences Centre and University of Toronto, Bayview Avenue, Toronto, ON, M4N 3M5, Canada

## Abstract

**Introduction:**

This systematic review looks at the use of noninvasive ventilation (NIV), inclusive of noninvasive positive pressure ventilation (NPPV) and continuous positive pressure ventilation (CPAP), in patients with chest trauma to determine its safety and clinical efficacy in patients with blunt chest trauma who are at high risk of acute lung injury (ALI) and respiratory failure.

**Methods:**

We searched the MEDLINE, EMBASE and Cochrane Central Register of Controlled Trials (CENTRAL) databases. Pairs of reviewers abstracted relevant clinical data and assessed the methodological quality of randomized controlled trials (RCTs) using the Cochrane domain and observational studies using the Newcastle-Ottawa Scale.

**Results:**

Nine studies were included (three RCTs, two retrospective cohort studies and four observational studies without a comparison group). There was significant heterogeneity among the included studies regarding the severity of injuries, degree of hypoxemia and timing of enrollment. One RCT of moderate quality assessed the use of NPPV early in the disease process before the development of respiratory distress. All others evaluated the use of NPPV and CPAP in patients with blunt chest trauma after the development of respiratory distress. Overall, up to 18% of patients enrolled in the NIV group needed intubation. The duration of NIV use was highly variable, but NIV use itself was not associated with significant morbidity or mortality. Four low-quality observational studies compared NIV to invasive mechanical ventilation in patients with respiratory distress and showed decreased ICU stay (5.3 to 16 days vs 9.5 to 15 days), complications (0% to 18% vs 38% to 49%) and mortality (0% to 9% vs 6% to 50%) in the NIV group.

**Conclusions:**

Early use of NIV in appropriately identified patients with chest trauma and without respiratory distress may prevent intubation and decrease complications and ICU length of stay. Use of NIV to prevent intubation in patients with chest trauma who have ALI associated with respiratory distress remains controversial because of the lack of good-quality data.

## Introduction

Management of patients with blunt chest trauma focuses on interventions such as the stabilization of fractures, pulmonary toilet, effective physiotherapy, and early and adequate pain control [1,2] These patients are at high risk for developing respiratory failure [3], with reports of up to 20% of patients with blunt chest trauma developing acute lung injury (ALI) or acute respiratory distress syndrome (ARDS) [1]. Intubation rates range from 23% to 75% and depend on the severity of the trauma, the degree of the underlying lung disease, and the intensity of initial management and monitoring [1,4]. The use of positive pressure ventilation has decreased the overall morbidity and mortality associated with blunt chest trauma [3], but endotracheal intubation and mechanical ventilation is associated with a high risk of nosocomial pneumonia and prolonged mechanical ventilation [5].

The role of noninvasive ventilation (NIV), which we consider to be either continuous positive airway pressure (CPAP) or noninvasive positive pressure ventilation (NPPV), for the management of patients with blunt chest trauma has not been established [5,6]. Although the safety of both CPAP and NPPV has been assessed in a number of observational studies in patients with blunt thoracic injuries [7-10], the evidence regarding the use of NIV in this setting is inconsistent [6]. Data derived from large multicenter trials evaluating NIV use in hypoxemic patients is not generalizable to these patients, as these trials included few trauma patients [11]. Two recent guidelines have offered a "no recommendation" or a "low-grade recommendation" for the use of NIV in blunt chest trauma [12,13]. However, these guidelines do not include the totality of the available data for this clinical condition.

The objective of this systematic review was to assess the current evidence regarding the use of NIV for patients with blunt thoracic trauma, to identify the most appropriate time to implement NIV and the safety of its use.

## Methods

### Data sources, searches and study selection

We searched the MEDLINE (1946 through June 2012), EMBASE (1980 through June 2012) and Cochrane Central Register of Controlled Trials (CENTRAL) databases using the search terms for NIV and blunt chest trauma (see Additional File 1 for the complete search strategy). Reference lists of retrieved articles and personal files were also searched.

We included published studies in any language, regardless of study design, that reported on clinical outcomes (for example, rate of endotracheal intubation, mortality) in patients with blunt chest trauma who were managed with NIV. There were no age restrictions. We also included case series and cohorts with no comparison groups to look at the safety of NIV in this patient setting. We excluded case reports, qualitative studies and economic analyses.

For the purpose of this systematic review, we defined *noninvasive ventilation *as the use of any degree of positive end-expiratory pressure or pressure support applied by facemask, helmet mask or nasal prongs. Thus, studies using CPAP or NPPV were included. *Blunt chest trauma *was defined as the presence of pulmonary contusions, rib fractures and flail chest or sternal fractures. The severity of injury was evaluated based on the Injury Severity Score (ISS) or the Thoracolumbar Injury Classification and Severity Score [14,15]. We ascertained the presence of acute hypoxemia using the partial pressure of arterial oxygen to the fraction of inspired oxygen ratio (PaO_2_:FiO_2_) of 300 mmHg or less (for ALI) or 200 mmHg or less (for ARDS) (16). Two investigators (AD and PP) independently and in duplicate completed the literature search and located potentially eligible articles.

### Data extraction and quality assessment

Two investigators (AD and PP) independently extracted the following data: authors, year and country of publication, ICU type, study design, inclusion and exclusion criteria, number of patients included, severity of hypoxemia and severity of injury (chest and overall trauma). We reviewed clinically relevant outcomes. The primary outcome of interest was the duration of ventilation in patients undergoing NIV compared to mechanical ventilation. Secondary outcomes included in-hospital mortality, ICU and hospital length of stay, development of nosocomial infections and development of any barotrauma. We assessed the use of NIV for ventilatory support in the patients who developed hypoxemic respiratory failure and ARDS and compared it with endotracheal intubation and mechanical ventilation. We also looked at the use of NIV compared to high-flow oxygen through facemask to determine the need for mechanical ventilation. We extracted safety data, including rate of NIV failure, associated mortality, nosocomial infection and barotrauma.

To assess the methodological quality of the included randomized controlled trials (RCTs), we followed the recommendations outlined in the *Cochrane Handbook for Systematic Reviews of Interventions *(domain-based evaluation of seven components) [17]. To assess the methodological quality of the included observational studies, we used the Newcastle-Ottawa Scale (point-based evaluation of the eight components). We modified the Newcastle-Ottawa Scale to look at the methodological quality of the case series and cohort studies without comparison groups by developing a six-point scale [18].

### Data synthesis

We could not combine the data from any of the studies because of the clinical heterogeneity that existed during the time period of the intervention as well as the differences in patient selection criteria, severity of injury and comparison groups.

## Results

### Study selection

**Figure 1:**
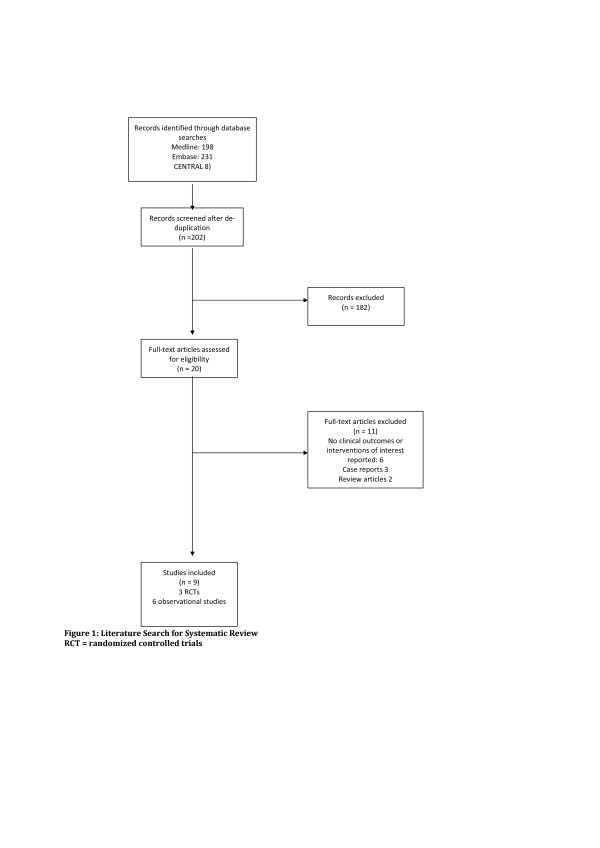
**Literature search for systematic review**.

The initial search strategy identified 20 potentially eligible studies (Figure 1). We excluded 11 studies for the following reasons: six studies did not report any clinical outcomes, three were case reports and two were review articles (Additional File 2). Nine studies met our inclusion criteria. Three were RCTs [6,20,21], two were retrospective cohort studies [5,19] and four were observational studies [7-10].

### Study characteristics

We report the main characteristics of the nine included studies in Table 1. Three main groups of studies were included. (1) Hernandez *et al*. evaluated early use of NIV as compared to high-flow oxygen in patients with hypoxemia with or without signs of any respiratory distress within the first 8 h of presentation to the emergency room or ICU [6]. (2) Four studies evaluated patients late in their course of respiratory failure [5,19-21]. These studies, in which NIV was introduced following the development of respiratory distress, compared the use of NIV to prevent intubation with early intubation. (3) Another four studies, which did not include a comparator group, assessed the safety of CPAP and NPPV after respiratory distress had developed in the study participants [7-10]. Accordingly, owing to the differences in the timing of initiation of NIV and the focus on patient safety, we classified these studies into early interventions, late interventions and patient safety assessment categories (Table 1). Analgesia was an integral part of the initial management of these patients. Eight of the studies mentioned the strategies for pain control used in conjunction with NIV (Table 1).

**Table 1:** Study characteristics.^a^

Study (design)	Patients (*n*)/country	Study intervention	Control intervention	Severity of hypoxemia	Quantification of severity of chest injury	Strategy for pain control
I. Early interventions: CPAP/NPPV compared to supplemental oxygen						

Hernandez *et al*. (2010) (RCT)	50/Spain	NPPV	High-flow oxygen	PaO_2_/FiO_2 _≤200 for >8 h	Thoracic AIS, ISS, lung contusions/quadrant, thoracolumbar vertebral trauma, flail chest	Epidural analgesia (bupivacaine and fentanyl) Remifentanil infusion



II. Late interventions: CPAP/NPPV after development of respiratory distress						

Xirouchaki *et al*. (2004) (case series)	22/Greece	NPPV	None	PaO_2_/FiO_2 _≤140	AIS, ISS	Epidural analgesia IV analgesia (not specified)

Tanaka *et al*. (2001) (case series)	59/Japan	CPAP	None	Not Reported	AIS, ISS, flail chest	Epidural analgesia (not specified)

Walz *et al*., 1998 (case series)	30/Germany	CPAP	None	PaO_2 _≤70	Isolated or accompanying chest trauma on one or both sides	Epidural analgesia Intercostal nerve blocks IV analgesia

Hurst *et al*. (1985) (case series)	33/USA	CPAP	None	PaO_2_/FiO_2 _<150 or PaO_2 _<65	Chest trauma (lung contusions, rib fractures) No severity score used	Not specified

III. Patient safety assessment: CPAP/NPPV compared to mechanical ventilation after development of respiratory distress						

Gunduz *et al*. (2005) (RCT)	43/Turkey	CPAP	Mechanical ventilation	PaO_2_/FiO_2 _≤300	TTSS, five or more rib fractures in a row, three or more segmental rib fractures, flail chest	Morphine patient Controlled analgesia

Bolliger and Van Eeden (1990) (RCT)	69/South Africa	CPAP	Mechanical ventilation	PaO_2_/FiO_2 _≥150	ISS, more than three rib fractures, pulmonary contusion	Lumbar epidural analgesia (buprenorphine) Intercostal nerve blocks

Vidhani *et al*. (2001) (retrospective cohort)	75/Australia	NPPV	Mechanical ventilation	PaO_2 _<65 or PaO_2_/FiO_2 _≤300	ISS, unilateral or bilateral lung contusion, flail chest	Epidural analgesia Patient-controlled analgesia Oral analgesics

Linton *et al*. (1982) (retrospective cohort)	26/South Africa	CPAP	Mechanical ventilation	PaO_2_/FiO_2 _≥150	Number of rib fractures, bilateral rib fractures, flail chest	Epidural analgesia (buprenorphine and morphine)

^a^AIS: Abbreviated Injury Score; CPAP, continuous positive airway pressure; ISS: Injury Severity Score; IV, intravenous; NPPV: noninvasive positive pressure ventilation; PaO_2_/FiO_2_: partial pressure of oxygen to fraction of inspired oxygen ratio; RCT: randomized controlled trial; TTSS: Thorax Trauma Severity Score.

### Study quality

Among the three RCTs, only one [6] was of moderate quality; the other two [20,21] were of low quality because of concerns regarding blinding, allocation and reporting of outcomes (Table 2). The two retrospective cohort studies were considered to be of low quality because of concerns regarding the assessment of outcomes and follow-up in the cohort studies (Table 3). Because of the high risk of selection bias due to the lack of a comparison group and important shortcomings in their reporting of outcomes, we considered observational studies without a comparison group to be of low quality (Table 4).

**Table 2:** Quality assessment of randomized controlled trials

	Selection bias		Performance bias	Detection bias		Attrition bias	Reporting bias
**Study**	**Random sequence generation**	**Allocation concealment**	**Blinding of participants and personnel**	**Blinding for patient reported outcomes**	**Blinding for clinical outcomes (mortality)**	**Incomplete outcome data**	**Selective reporting**

Hernandez *et al*. (2010)	Low	Low	Uncertain	Uncertain	Uncertain	Low	Low

Gunduz *et al*. (2005)	High	High	High	High	High	Uncertain	Uncertain

Bolliger and Van Eeden (1990)	High	High	High	High	High	Uncertain	Uncertain

^a^Studies were considered to be of moderate quality if there were more than two domains with uncertain risk of bias. They were considered to be of low quality if four or more domains had uncertain risk of bias or if one domain had a high risk of bias.

**Table 3:** Quality assessment of observational studies

		Selection		Comparability		Outcome/exposure			
**Study**	**Representation of the exposed cohort**	**Selection of non exposed cohort**	**Ascertainment of exposure**	**Outcome not present at start**	**Comparability of cohorts based on design or analysis**	**Assessment of outcomes**	**Time for follow-up**	**Adequacy of follow-up**	**Overall score**

Linton *et al*. (1982)	*	*	*	*	-	*	-	-	Moderate

Vidhani *et al*. (2001)	*	-	*	*	*	_	_	-	Low

^a^Asterisks in each category signify identification of an appropriate methodological process in the study. Studies were considered to be of high quality if less than two components of the Newcastle-Ottawa Scale were missing, fair quality if two or three components were missing and low quality if more than three components were missing.

**Table 4:** Quality assessment of observational studies with no comparison group.^a^

	Selection			Exposure/outcome			Overall score
**Study**	**Representation of the exposed cohort**	**Ascertainment of exposure**	**Outcome not present at start**	**Assessment of outcomes**	**Time for follow-up**	**Adequacy of follow-up**	

Xirouchaki *et al*. (2004)	*	*	*	-	-	-	Low

Tanaka *et al*. (2001)	*	*	_	_	-	-	Low

Walz *et al*. (1998)	*	*	*	-	-	-	Low

Hurst *et al*. (1985)	*	*	-	-	-	-	Low

^a^Asterisks in each category signify identification of an appropriate methodological process in the study. Studies were considered to be of low quality if any component of the Newcastle-Ottawa Scale was missing, as there was no comparison group, which introduces selection bias at baseline.

### Safety of noninvasive ventilation in patients with blunt chest trauma

Table 5 reports the rates of failure of NIV, nosocomial pneumonia, barotrauma and mortality associated with NIV. Four studies assessed the rate of intubation in patients undergoing treatment with NIV at any time during their hospital admission [6,7,10,20]. The reported rate of NIV failure ranged from 12% to 18%. Hurst *et al*. found a much lower rate of intubation (6%), but the severity of chest injury in their patients was very low and was not comparable to that reported in the other studies [10]. Gunduz *et al*. reported no need for intubation in patients undergoing NIV but excluded patients undergoing emergent intubation after randomization from their analysis [20]. If those patients had been included, the rate of intubation would have been 17.1% [20].

**Table 5:** Outcomes associated with noninvasive ventilation.^a^

Study (design)	Need for intubation due to failure of noninvasive ventilation	Nosocomial infection	Pneumothorax	Mortality
Hernandez *et al*. (2010) (RCT)	12% in NIV vs 40% in high-flow oxygen group	8% in NIV vs 12% in high-flow oxygen group	24% in NIV vs 12% in high-flow oxygen group	4% in NIV vs 4% in high-flow oxygen group

Gunduz *et al*. (2005) (RCT)	17%*	9%	-	9%

Xirouchaki *et al*. (2004) (case series)	18%	13.6%**	-	0

Vidhani *et al*. (2001) (retrospective cohort)	-	-	-	0

Tanaka *et al*. (2001) (case series)	-	-	-	-

Walz *et al*. (1998) (case series)	-	-	-	0

Bolliger and Van Eeden (1990) (RCT)	-	13.8%	5.5%	0

Hurst *et al*. (1985) (case series)	6%	-	-	-

Linton *et al*. (1982) (retrospective cohort)	-	0	-	-

^a^NIV, noninvasive ventilation. Data are reported as rates (%) for all variables. *The rate of intubation was not reported in the journal text, as patients needing emergent intubation were excluded from the analysis. **All nosocomial pneumonia occurred in patients who required intubation after initial management with NIV.

Nosocomial pneumonia and pneumothorax were the most commonly reported adverse events associated with NIV use. Nosocomial pneumonia was reported in five studies, and the rate ranged from 8% to 13.8% [5-7,20,21]. The rate of pneumothorax reported in two studies ranged from 5.5% to 24% [6,21]. Of note, the overall event rate for these two aforementioned events was extremely low. Only two studies reported any deaths associated with the use of NIV, which were also low at 4% and 9% [6,20].

### Noninvasive ventilation compared to high-flow oxygen

A single study compared NIV to the use of high-flow oxygen therapy (≥10 L/min) [6]). In this study, patients with ALI and blunt chest trauma were randomized to early NIV or high-flow oxygen therapy. Both groups had similar ISSs and degrees of hypoxemia. The utilization of cointerventions, such as epidurals and patient-controlled analgesia (PCA) for pain control, early chest physiotherapy and need for surgical intervention, was also similar in both groups. Although the study was stopped early, the use of NIV was associated with a lower rate of endotracheal intubation (3 (12%) of 25 patients vs 10 (40%) of 25 patients; *P *= 0.02). The need for intubation occurred within the first 24 hours in all the patients in the high-flow oxygen group and after 72 hours in all the patients in the NIV group. There were no differences in the rate of pneumothorax, ICU length of stay and mortality.

## Noninvasive ventilation compared to endotracheal intubation and mechanical ventilation

Four studies compared NIV to endotracheal intubation [7-10]. Three studies used CPAP [8-10], and one study used NPPV [7] (Table 6). All of these studies had methodological limitations (Tables 2 through 4). Mortality, ICU length of stay and the rates of nosocomial pneumonia were much higher in the intubated patients compared to patients undergoing NIV (Table 6).

**Table 6:** Outcomes in patients receiving continuous positive pressure ventilation and/or noninvasive positive pressure ventilation compared to mechanical ventilation

Study (design)	Duration of mechanical ventilation		Mortality		Length of stay in ICU/hospital		Nosocomial pneumonia	
	CPAP/NPPV	Mechanical ventilation	CPAP/NPPV, *n *(%)	Mechanical ventilation, *n *(%)	CPAP/NPPV	Mechanical ventilation	CPAP/NPPV, *n *(%)	Mechanical ventilation, *n *(%)

Gunduz *et al*. (2005) (RCT)	15 ± 4 days	-	2/22 (9%)	7/21 (33%)	16 (3)	15 (4)	2/22 (9%)	10/21 (47%)

Bolliger and Van Eeden (1990) (RCT))	4.5 ± 2.3 days	7.3 ± 3.7 days	0/36 (0)	2/33 (6%)	5.3 ± 2.9 days	9.5 ± 4.4 days	5/36 (14%)	16/33 (49%)

Vidhani *et al*. (2001) (retrospective cohort)	-	-	0/12 (0)	14/28 (50%)	7 (3 to 26)	-	-	-

Linton *et al*. (1982) (retrospective cohort)	-	-	-	-	7 (3 to 21)	12 (7 to 120)	0	5/13 (38%)

^a^CPAP: Continuous Positive Airway Pressure; NPPV: Noninvasive Positive Pressure Ventilation; RCT: randomized controlled trial. Data are reported as means ± standard deviation or medians with interquartile range for continuous variables and rates (%) for all other variables.

Comparisons between the NIV patients and those who were mechanically ventilated were not possible because of important differences in ISS between the two groups. The rates of ISSs and rates of neurological injuries were different in the two groups in studies conducted by Bolligher and Van Eeden [21], Vidhani *et al. *[19] and Linton *et al. *[5]. In these studies, patients with severe injuries or decreased levels of consciousness required mechanical ventilation compared to patients with less severe injuries who were treated with NIV. The differences in the need for transfusion of blood products in either of the groups were not mentioned in any of the studies.

## Discussion

Our systematic review assessed the use of NIV for patients with blunt chest trauma. Studies included in our review ranged from moderate (one RCT) to low quality. We found that the application of this modality was highly variable in clinical practice [6,21,22]. NIV was instituted mostly when patients had already developed acute respiratory decompensation associated with hypoxemia. Moreover, the timing of initiation of NIV was variable, ranging from a few hours to a few days after hospital admission. In the single RCT, which instituted NIV prior to the development of respiratory failure, intubation rates were low in the patients who had moderate ISSs [6]. The lower rate of intubation in this study may have been due to the early institution of NIV. None of the studies compared CPAP to NPPV. On the basis of the findings of our review, both modalities seem to be safe for use in appropriate patients with blunt chest trauma.

Although there are low- to moderate-quality data on the use of NIV in blunt chest trauma, there may be a role, albeit limited, for the early use of NIV in patients with blunt chest trauma. The RCT data reported by Hernandez *et al*. suggests that early identification of at-risk patients with prompt institution of NIV in appropriate patients may be of greatest benefit because their NIV failure and mortality rates were lower than those found in the studies where NIV was initiated following the development of respiratory failure [6].

Patients who develop hypoxemic failure later in the course of their hospitalization likely have other factors present, such as progression of lung contusions or the development of pneumonia or ARDS, that result in severe hypoxemia and respiratory distress. Animal studies have shown that lung contusions and associated areas of rib fractures reduce lung compliance, increase shunt fraction and cause capillary leak in the injured and uninjured lung [24,25]. These pathophysiological findings explain the high likelihood of hypoxemic respiratory failure and potential of progression to ARDS in these patients. The rate of failure of NIV in patients with blunt chest trauma who developed acute respiratory distress and respiratory failure in the studies included in our review was very close to the rates reported by Antonelli *et al*. for any cause of acute hypoxemic respiratory failure [11,23]. There is clear evidence that NIV has a limited role in patients with hypoxemic respiratory failure due to ARDS or infection and in fact might be detrimental [13,26]. Similarly, our review suggests that a clear role for the use of NIV in blunt thoracic trauma remains uncertain.

When considering a trial of NIV in patients with blunt chest trauma, NIV should be initiated for 48 to 72 h. After the initial stabilization of patients, failure of NIV has been reported mostly following this period [6]. In addition, most of the safety data on the use of NIV derived from observational studies refers to its use within the first 48 to 72 hours after trauma. Thus, for patients who are unresponsive to NIV, NIV should be discontinued as soon as possible within the first 24 hours and endotracheal intubation should be considered early to mitigate the potential for harm.

Length of stay in ICU was lower in patients with NIV use compared to invasive mechanical ventilation [5,20,21]. In all studies, however, the ventilated patients received continuous sedation, whereas the NIV group received either epidural anesthesia or PCA. As these studies did not use spontaneous breathing trials and sedation interruption, the duration of endotracheal intubation was most likely a major driver of the length of stay in the ICU [27].

A major component of care for patients with blunt chest trauma is the need for adequate pain control. There is convincing evidence for the use of early epidurals, nerve blocks or PCA pumps for these patients [28]. All of these studies judiciously used early epidurals with good pain control along with the use of NIV. In situations where epidurals were not possible or contraindicated, analgesia with nerve blocks and PCA was instituted [6,7,20,21]. Unfortunately, this aspect of care for these patients is often overlooked. A close working relationship with the Anesthesia and Pain Control Service might improve the institution of NIV when a patient with blunt chest trauma is being evaluated.

We did not identify any significant morbidity or mortality associated with the use of NIV in patients with blunt chest trauma. Even though the reported mortality was higher in patients undergoing invasive mechanical ventilation compared to NIV, most patients died as a result of their injuries and not as a direct result of respiratory failure. These data reaffirm the need for proper patient selection and the continuous close monitoring of patients being treated with NIV. These patients' conditions can deteriorate very quickly, and their respiratory and overall clinical status should be reassessed often when they are undergoing treatment with NIV.

There are a number of limitations of this systematic review. Studies have reported very heterogeneous clinical data in this field, which is difficult to interpret. We acknowledge that most studies included in this review looked at different modalities, different time periods and also, in some regards (that is, ALI severity and ISSs), different patients. We also have to acknowledge that the progression of respiratory failure may be dependent on nonthoracic injuries and other factors such as transfusion-associated reactions. Even though the studies performed by Bolliger and Van Eeden [21] and Linton *et al. *[5] had higher ISSs in the intubated patients, there were no differences in the ISSs and trauma patterns between the different groups. We feel, however, that it is critical to highlight this heterogeneity so that clinicians are careful in the way they apply this intervention to patients with blunt chest trauma. It is also important that these limitations are addressed in any future studies addressing this clinical question.

Our study has a number of strengths. We grouped the studies based on the timing of the intervention and the clinical severity of disease. This approach provides a more clear understanding of the utilization of NIV in this population and also highlights the potential pitfalls of using this intervention inappropriately. Our review suggests that the benefit of NIV is early in the course of blunt chest trauma, prior to the development of overt respiratory failure, and that prompt, appropriate institution of this modality can prevent endotracheal intubation. We are able to reaffirm that NIV should be used only in specialized settings by institutions with adequate expertise to handle any complications arising from initiating NIV.

## Conclusion

On the basis of the findings of our review, NIV may be considered in patients with blunt chest trauma who are neurologically intact, hemodynamically stable and not in respiratory distress. There is no apparent benefit of NIV in the prevention of intubation in patients with respiratory decompensation. In fact, delaying intubation in these patients leads to harm. Future studies need to be methodologically sound and focus on the use of NIV in patients with blunt chest trauma early in the course of the disease, prior to overt respiratory failure.

## Key messages

• NIV may be considered in patients with blunt chest trauma who are neurologically intact, hemodynamically stable and not in respiratory distress.

• There is no apparent benefit of NIV in the prevention of intubation in patients with respiratory decompensation.

• NIV should be used only in specialized settings by institutions with adequate expertise to handle any complications arising from initiating NIV.

• The benefit of NIV is early in the course of blunt chest trauma, prior to the development of overt respiratory failure.

• A well-designed, methodologically sound RCT is needed to assess the role of NIV in blunt chest trauma.

## Abbreviations

ALI: acute lung injury; ARDS: acute respiratory distress syndrome; CPAP: continuous positive airway pressure; NIV: noninvasive mechanical ventilation; NPPV: noninvasive positive pressure ventilation; RCT: randomized controlled trial

## Competing interests

This study was not funded. None of the authors have any personal or financial support or involvement with any organization with financial interest in the subject matter or any actual or potential conflict of interest.

## Authors' contributions

AD was involved in the conception and design of the study, analysis of the data and drafting of the article. PP and EG were involved in analysis of the data and revision of the manuscript. TS was involved in the conception of the study and drafting of the article. LT was involved in the design of study, drafting of the article and critical revision of the manuscript. All authors approved the final version of the manuscript.

## Supplementary Material

Additional File 1 Supplementary File 1: Search Strategy Click here for file

Additional Files 2 Supplementary File 2: Excluded studies Click here for file
