# Supplementary material for: Safety and efficacy of noninvasive ventilation in patients with blunt chest trauma: a systematic review
Source: Crit Care. 2013 Jul 22;17(4):R142. doi: 10.1186/cc12821 (PMC4057415; doi:10.1186/cc12821)
Supplement: Additional File 1 — Supplementary File 1: Search Strategy [file cc12821-S1.DOC]

**Supplementary File 1: Search Strategy for MEDLINE and CENTRAL, and was appropriately modified for EMBASE**

1. exp *Respiration, Artificial/

2. exp *Continuous Positive Airway Pressure/

3. Continuous positive airway$.mp. [mp=ps, rs, ti, ot, ab, nm, hw, ui, sh, tn, dm, mf, dv, kw, tx, ct]

4. Biphasic intermittent positive airway.mp.

5. Bilevel positive airway$.mp.

6. Noninvasive ventilatory-assistance apparatus.mp.

7. Noninvasive support ventilation.mp.

8. Noninvasive ventilat$.mp. [mp=ps, rs, ti, ot, ab, nm, hw, ui, sh, tn, dm, mf, dv, kw, tx, ct]

9. Non-invasive ventilat$.mp. [mp=ps, rs, ti, ot, ab, nm, hw, ui, sh, tn, dm, mf, dv, kw, tx, ct]

10. Non-invasive positive pressure ventilation.mp.

11. Noninvasive positive pressure ventilation.mp.

12. Non-invasive ventilation.mp.

13. Noninvasive ventilation.mp.

14. Nasal ventilation.mp.

15. exp *Positive-Pressure Respiration/

16. exp *Ventilators, Mechanical/

17. Bi-level Positive Airway Pressure.mp.

18. BIPAP.mp.

19. exp *Intermittent Positive-Pressure Ventilation/

20. Intermittent positive pressure ventilation.mp.

21. exp *Positive-Pressure Respiration/

22. Positive pressure ventilation.mp. [mp=ps, rs, ti, ot, ab, nm, hw, ui, sh, tn, dm, mf, dv, kw, tx, ct]

23. Bilevel.mp. [mp=ps, rs, ti, ot, ab, nm, hw, ui, sh, tn, dm, mf, dv, kw, tx, ct]

24. Pressure support.mp.

25. Non-invasive.mp.

26. CPAP.mp.

27. 1 or 2 or 3 or 4 or 5 or 6 or 7 or 8 or 9 or 10 or 11 or 12 or 13 or 14 or 15 or 16 or 17 or 18 or 19 or 20 or 21 or 22 or 23 or 24 or 25 or 26

28. chest trauma.mp. [mp=ps, rs, ti, ot, ab, nm, hw, kw, ui, an, sh, tn, dm, mf, dv, tx, ct]

29. blunt chest trauma.mp. [mp=ps, rs, ti, ot, ab, nm, hw, kw, ui, an, sh, tn, dm, mf, dv, tx, ct]

30. flail chest.mp. [mp=ps, rs, ti, ot, ab, nm, hw, kw, ui, an, sh, tn, dm, mf, dv, tx, ct]

31. lung trauma.mp. [mp=ps, rs, ti, ot, ab, nm, hw, kw, ui, an, sh, tn, dm, mf, dv, tx, ct]

32. pulmonary trauma.mp. [mp=ps, rs, ti, ot, ab, nm, hw, kw, ui, an, sh, tn, dm, mf, dv, tx, ct]

33. lung contusion.mp. [mp=ps, rs, ti, ot, ab, nm, hw, kw, ui, an, sh, tn, dm, mf, dv, tx, ct]

34. pulmonary contusion.mp. [mp=ps, rs, ti, ot, ab, nm, hw, kw, ui, an, sh, tn, dm, mf, dv, tx, ct]

35. thoracic injury.mp. [mp=ps, rs, ti, ot, ab, nm, hw, kw, ui, an, sh, tn, dm, mf, dv, tx, ct]

36. blunt thoracic trauma.mp. [mp=ps, rs, ti, ot, ab, nm, hw, kw, ui, an, sh, tn, dm, mf, dv, tx, ct]

37. thoracic injury.mp. [mp=ps, rs, ti, ot, ab, nm, hw, kw, ui, an, sh, tn, dm, mf, dv, tx, ct]

38. 28 or 29 or 30 or 31 or 32 or 33 or 34 or 35 or 36 or 37

39. 27 and 38
