# Supplementary material for: Safety and efficacy of noninvasive ventilation in patients with blunt chest trauma: a systematic review
Source: Crit Care. 2013 Jul 22;17(4):R142. doi: 10.1186/cc12821 (PMC4057415; doi:10.1186/cc12821)
Supplement: Additional Files 2 — Supplementary File 2: Excluded studies [file cc12821-S2.DOCX]

**Supplementary File 2: Characteristics of Excluded Studies**

| **Study, year of publication** | **Citation** | **Reason for exclusion** |
| --- | --- | --- |
| Condon et al, 2011 | Applied Cardiopulmonary Pathophysiology 2011; 15: 51-54, | Case Report |
| Reid et al, 1965 | Br Med J. 1965 Apr 24; 1 (5442): 1105-9. | Intervention relevant to study not present |
| Craven et al, 1979 | J Appl Physiol. 1979 Oct; 47 (4): 729-37. | Outcomes relevant to study not reported |
| Garfield et al, 2000 | Br J Anaesth. 2000 Nov; 85 (5): 788-90. | Case Report |
| Simon et al, 2006 | EAST Guidelines | Review |
| Gregoretti et al, 1998 | Intensive Care Med (1998) 24:785-790 | Outcomes relevant to study not reported |
| Al Ansari et al, 2006 | Saudi Med J. 2006 Aug; 27(8): 1244-7. | Case Report |
| Pettiford et al, 2007 | Thorac Surg Clin. 2007 Feb; 17(1): 25-33 | Review |
| Schweiger et al, 2003 | Crit Care Med. 2003 Sep;31(9):2364-70. | Outcomes relevant to study not reported |
| Bourvine et al, | Harefuah. 1991 Nov 1;121(9):302-5 | Intervention relevant to study not present |
| Fiandri et al | Minerva Anestesiol. 1990 Oct;56(10):1251-2. | Outcomes relevant to study not reported |
